# Environmentally Friendly Fluoroquinolone Derivatives with Lower Plasma Protein Binding Rate Designed Using 3D-QSAR, Molecular Docking and Molecular Dynamics Simulation

**DOI:** 10.3390/ijerph17186626

**Published:** 2020-09-11

**Authors:** Yilin Hou, Yuanyuan Zhao, Yu Li

**Affiliations:** 1 College of Environmental Science and Engineering, North China Electric Power University, Beijing 102206, China; hylshinanshen95@outlook.com (Y.H.); zyy950210@outlook.com (Y.Z.); 2 MOE Key Laboratory of Resources and Environmental System Optimization, College of Environmental Science and Engineering, North China Electric Power University, Beijing 102206, China

**Keywords:** fluoroquinolone, plasma protein binding rate, three-dimensional quantitative structure–activity relationship, molecular modification, molecular docking, molecular dynamics simulation

## Abstract

Comparative molecular similarity index analysis (CoMSIA) was used to establish a three-dimensional quantitative structure–activity relationship (3D-QSAR) model with structural parameters of quinolones as the independent variables and plasma protein binding rate (log*f*_b_) as the dependent variable to predict the log*f*_b_ values of remaining quinolones in this study. In addition, the mono-substituted and bis-substituted reaction schemes that significantly influenced the plasma protein binding rate of quinolones were determined through an analysis of the 3D-QSAR contour maps. It was found that the replacement of small groups, hydrophobic groups, electronegative groups, or hydrogen bond acceptor groups at the substitution sites significantly reduce the log*f*_b_ values of quinolone derivatives. Furthermore, the mechanism of decrease in binding rate between trovafloxacin (TRO) derivatives and plasma protein was revealed qualitatively and quantitatively based on molecular docking and molecular dynamics simulation. After modification of the target molecule, 11 TRO derivatives with low plasma protein binding rates were screened (reduced by 0.50–24.18%). Compared with the target molecule, the molecular genotoxicity and photodegradability of the TRO derivatives was higher (genotoxicity increased by 4.89–21.36%, and photodegradability increased by 9.04–20.56%), and their bioconcentration was significantly lower (by 36.90–61.41%).

## 1. Introduction

Quinolones are synthetic antibacterial agents developed from nalidixic acid [1], and they share a common structure with pyridone acid [2]. Since the 1980s, the production and utilization of fluoroquinolones have increased rapidly because of their good pharmacokinetic properties, broad antibacterial spectrum, strong antibacterial activity, high bioavailability and long half-life. Their metabolization is relatively slow. Thus, they have become the most widely used antibacterial agents in clinical practice [3]. However, this widespread use of quinolones has led to a series of toxic side effects on organisms [4].

Studies have shown that when drugs are ingested, they bind to plasma proteins in the blood and form drug-protein complexes [5]. As antigens, fluoroquinolones acquire immunogenicity when combined with plasma proteins, thus stimulating the body to produce a drug-antigen-specific antibody. This antibody binds to a drug-bound leukocyte or to a drug to form an antigen–antibody complex, which subsequently binds to leukocytes with an IgG Fe (Immunoglobulin G-Fe, IgG Fe) receptor to cause leukopenia [6]. Therefore, for reliable drug design, scientific prediction of the functional and environmentally friendly parameters of compounds is required. In computer-aided drug design, known knowledge about drug molecules and biological target cells are used to discover and design novel drug molecules through theoretical simulation and calculation [7]. Although the rate at which drugs bind to plasma protein can be measured with experimental methods such as ultrafiltration, equilibrium dialysis, and microdialysis [8], such measurements generally require complicated processes and often are expensive. Therefore, it is very important to estimate or to predict the plasma protein binding rate of drugs by using a theoretical calculation method for rational screening and design of novel drugs.

In this study, we selected quinolone antibiotics as the research object and we used the three-dimensional quantitative structure–activity relationship (3D-QSAR) model to construct a CoMSIA module. Therefore, we used partial least squares regression analysis to establish the quantitative relationship between the plasma protein binding rate (log*f*_b_) of quinolones and their structure. With this model, we estimated the log*f*b values of 16 quinolones derivatives. According to the contour maps of quinolone (template molecule), the modified mono-substituted and bis-substituted sites were selected accurately, and trovafloxacin (TRO) was selected as the template molecule; thus, 11 types of TRO derivatives were screened. Then, we used the Gaussian 09 software to calculate the positive frequency of TRO derivatives and changes in the Gibbs free energy of the 11 substitution reactions. Thereafter, the function (plasma protein binding rate (Log*f*_b_), genotoxicity (negative logarithm of the Lowest Observed Effect Concentration, pLOEC)) and environmental-friendliness (bioconcentration (log*K*_ow_), photodegradability (log*t*_1/2_)) of the TRO derivatives were evaluated. The results indicated that TRO derivatives exhibited good stability (stable in the environment). While their genotoxicity and photodegradability increased, their bioconcentration decreased. Finally, changes with time in the trajectories of the complexes of trovafloxacin and its representative derivatives with plasma protein were simulated by molecular docking and molecular dynamics to verify and supplement microscopy data on the binding of quinolones to plasma protein. This study aimed to provide an applicable theoretical method for the research, prediction, and design of new quinolones with a lower plasma protein binding rate in clinical application and lower environmental damage.

## 2. Materials and Methods

### 2.1. Establishment of 3D-QSAR Model of Quinolones with Low Plasma Protein Binding Rate

3D-QSAR analysis was performed using the software SYBYL-X2.0 [9]. The plasma protein binding rate data (*f*_b_) of 16 quinolones were obtained by reference [10], of which 12 were fluoroquinolones. For convenience in QSAR analysis, the logarithms of fb (log*f*_b_) were considered as the indicators of the plasma protein binding rates of quinolones.

In order to calculate the parameters of the CoMSIA field, 16 log*f*_b_ values of the quinolones were successively input into the training table, and the parameters of the CoMSIA field were calculated automatically by SYBYL-X2.0 through autofill. Partial least squares (PLS) analysis was performed to establish a relationship between the structure and biological activity of the target compounds. In the PLS analysis, the compounds from the training set were cross-validated using the leave-one-out method to calculate the cross-validation correlation coefficient (*q*^2^) and the optimum number of components (*n*). Then, regression analysis was performed with no validation to calculate the non-cross-validation correlation coefficients (*r^2^*). Finally, the standard error of estimated (SEE) and test value (*F*) were calculated to complete the establishment of the CoMSIA model [11,12,13].

### 2.2. Evaluation of Functional Characteristics and Environmental-Friendliness of TRO Derivatives Using 3D-QSAR Model

The functional characteristics (plasma protein binding rate (log*f*_b_), genotoxicity (pLOEC)) and environmental friendliness (bioconcentration (log*K*_ow_), photodegradation (log*t*_1/2_)) of TRO derivatives were evaluated using the QSAR or hologram QSAR (HQSAR) method. The plasma protein binding rates (log*f*_b_) of the TRO derivatives were predicted using the 3D-QSAR model established in this article. The genotoxicity (pLOEC) of the TRO derivatives was predicted using the HQSAR model established by Zhao et al. [14] Bioconcentration (log*K*_ow_) and photodegradability (log*t*_1/2_) were predicted using the 3D-QSAR model established by Zhao et al. [15,16].

### 2.3. Interaction Characterisation between Fluoroquinolones and Plasma Protein Based on Molecular Dynamics Simulation

Molecular dynamics simulations were performed using the Standard Dynamics Cascade module in the software Discovery Studio (DS). The specific steps are the following:

(1) Initial structural examination and pre-treatment: The first steps in our molecular dynamics simulation were to determine the initial structure, to compute the initial position and initial velocity of each atom in the plasma protein structure, and to obtain plasma protein data from the protein database PDB (ID:5NU7).
(2) Simulation system field: The molecular force field was the basis of this molecular simulation. It was necessary to use the force field to reasonably constrain fluoroquinolone molecules before and after modification to achieve accurate simulation of its system in virtual space. The Chemistry at HARvard Macromolecular Mechanics (CHARMM) force field can obtain good results for various simulation systems, such as small molecules and solvated large biological systems [17].
(3) Solvent effect: A solvent environment was added to the dynamics simulation process to make it more realistic. Generally, physiological saline is the most frequently added solvent environment, therefore it was also chosen for this research.
(4) Minimization of initial structure: After initial system preparation, minimization of system energy was required to eliminate unreasonable molecular contact in the initial structure.
(5) Dynamics simulation: This simulation consisted of three main processes, heating, equilibration, and production. The system before and after modification with fluoroquinolone was gradually heated to reach the target temperature set in the system simulation. In the equilibration process, the main parameters such as changes in plasma protein structure and temperature were monitored, and the process provided support for system simulation. In the production process, the motion trajectories of individual particles were calculated according to the Newtonian mechanics theory and the predetermined interparticle interaction potential.

## 3. Results and Analysis

### 3.1. Establishment and Evaluation of Quinolone Plasma Protein Binding Rate 3D-QSAR Model

#### 3.1.1. Establishment of 3D-QSAR Model Based on Plasma Protein Binding Rate

**Figure 1:**
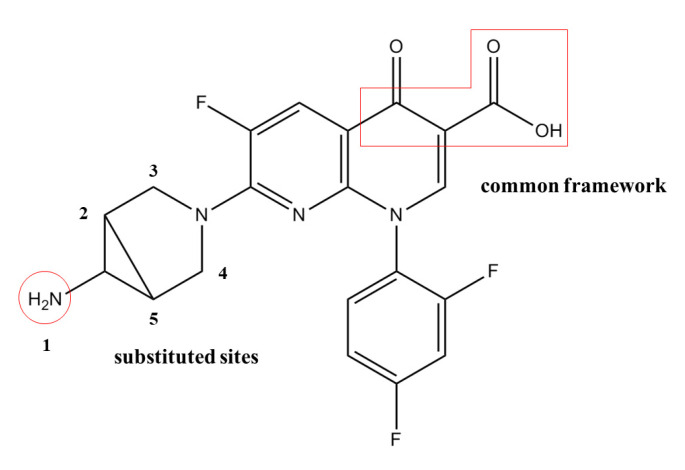
Molecular structure of trovafloxacin.

Log*f*_b_, the experimental value of the plasma protein binding rate of 16 quinolones, was used as the data source. Thirteen quinolones were randomly included in the training set, and the remaining 3 quinolones were included in the test set to establish the 3D-QSAR model. By using SYBYL-X2.0, the lowest-energy conformation of a molecule was selected as the dominant stable conformation, and the geometries of these compounds were subsequently optimized using the Tripos force field with Gasteiger–Hückel charges. Repeated minimizations were performed using the Powell method with the maximum number of iterations set to 10,000 to obtain an energy convergence gradient value of 0.005 kJ/mol [18,19]. The optimized molecules were stored in the database for alignment. By selecting the widely used trovafloxacin (TRO) as a template molecule, all molecules were aligned based on the pharmacophore characteristic elements as the common framework, as labelled in Figure 1. By using the CoMSIA module, a QSAR model suitable for predicting the plasma protein binding rate (log*f*_b_) of quinolones was established.

#### 3.1.2. Evaluation of 3D-QSAR Model of Quinolones’ Plasma Protein Binding Rate

The optimum number of components (*n*) of the CoMSIA model was found to be seven, and the cross-validated correlation coefficient *q*^2^ was 0.677, indicating the good predictive ability of the model (it is generally considered that a model has reliable predictive ability when *q*^2^ > 0.5) [20]. In addition, the non-cross-validation correlation coefficient *R^2^* was 0.998 (i.e., >0.9 and close to 1.0) [21,22]. *q*^2^ and *R*^2^ represent predictive ability and self-consistency. [23,24,25] SEE was 0.013, and test value (F) was 471.679, indicating the good fitting and predictive abilities of the CoMSIA model [26].

Golbraikh and Tropsha [27] confirmed that strict QSAR model validation procedures should include internal and external validation. The proposed model could not be evaluated using only internal validation parameters such as *q*^2^, so the external validation method was applied to evaluate the predictive ability of the proposed model. Moreover, the overall predictive ability of the CoMSIA model was verified externally by predicting the activity of the independent test set compounds. The predictive ability of the model is denoted by $r_{pred}^{2}$, and it can be calculated as follows:

(1) $$r_{pred}^{2}=1-\frac{\mathrm{PRESS}}{\mathrm{SD}}$$

where SD is the sum of the squares of the deviations between the experimental values of the test set compounds and the average of the experimental values of the compounds in the training set, and PRESS is the sum of the squared deviations between the experimental values and the predicted values of the compounds in the test set.

The activity of the test set was predicted using the established CoMSIA model (Table 1). According to the experimental values and those predicted using the CoMSIA model, the correlation coefficient of the test set prediction is 0.6879 (>0.6), indicating that the established 3D-QSAR model has a higher external prediction ability [28].

### 3.2. Molecular Modification of Fluoroquinolone with Low Plasma Protein Binding Rate Based on Contour Maps

In the CoMSIA model, the contributions of the steric field (S), electrostatic field (E), hydrophobic field (H), hydrogen bond donor field (D), hydrogen bond acceptor field (A) were respectively18.30%, 23.00%, 33.60%, 7.00%, and 18.10%. The results showed that logfb values of quinolones were affected by the spatial effect, electrical distribution, hydrophobicity of groups and hydrogen bond donors and receptors.

**Figure 2:**
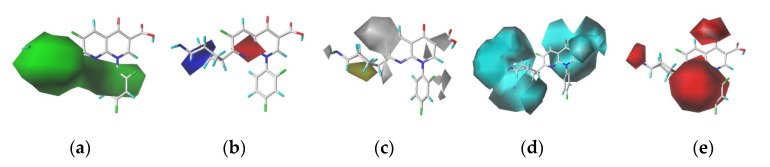
Contour maps of CoMSIA model, steric fields (**a**); electrostatic fields (**b**); hydrophobic fields (**c**); hydrogen-bond donor field (**d**); and hydrogen bond receptor field (**e**).

In this study, we selected trovafloxacin as the target molecule. In the contour maps generated using the CoMSIA model (Figure 2), the steric field represented by the green region is distributed near the 1-, 2-, 3-, 4-, and 5- substituents, indicating that the introduction of small groups at positions 1-, 2-, 3-, 4-, and 5- reduced the log*f*_b_ values of the quinolones and reduced their binding rates to plasma protein. The electrostatic field represented by the blue region is distributed near position 5-, indicating that the introduction of electropositive groups at position 5- could reduce the log*f*_b_ values of the quinolones. The white region representing the hydrophobic fields was distributed at the 1- substituent, indicating that the introduction of a hydrophobic group at position 1- could reduce the log*f*_b_ values of the quinolones. The hydrogen bond donor field represented by the cyan region is distributed at positions 1- and 2-, indicating that the introduction of the hydrogen bond donor at positions 1- and 2- could reduce the log*f*_b_ values of the quinolones. The red region representing hydrogen bond acceptors is distributed at the position 1- substituent, indicating that the introduction of the hydrogen bond acceptor at position 1- could reduce the log*f*_b_ values of the quinolones.

Comprehensive analysis of the contour maps obtained using the CoMSIA model revealed that position 1- was in the green, white, cyan, and red regions; position 2- was in the green and cyan regions; positions 3- and 4- were in the green region; and position 5- was in the green and blue regions. Position 1- was affected by the steric field, hydrophobic field, and hydrogen bond acceptor field. In other words, the introduction of small, hydrophobic, and hydrogen bond acceptor groups at this position was advantageous for reducing the log*f*_b_ values of the quinolones. Position 2- was influenced by the steric and hydrogen bond donor fields, and the introduction of small and hydrogen bond receptor groups at this site could reduce the log*f*_b_ values of the quinolones. Positions 3- and 4- were affected by the steric field, and the introduction of small groups at these positions proved beneficial for reducing the log*f*_b_ values of the quinolones. The introduction of small and electronegative groups at this position could reduce the log*f*_b_ values of the quinolones, on account of position 5- was affected by the steric and the electrostatic fields. Therefore, combining with the characteristics of trovafloxacin while not affecting its main structure, sites 1- and 5- were modified in this study to replace CH_3_ (site 1-) with five hydrophobic groups (-NO_2_, -C_2_H_5_, -C_3_H_7_, -SH and -OCH_3_) and H (site 5-) with two electronegative groups (-SiH_3_ and -PH_2_). Finally, a total of 16 TRO derivatives were produced (Table 2).

### 3.3. Evaluation of Functionality, Environmental-Friendliness, and Stability of TRO Derivatives Based on HQSAR and QSAR Models

#### 3.3.1. Functional Evaluation of TRO Derivatives Based on QSAR Model

The 3D-QSAR model of plasma protein binding rate constructed in this study and the HQSAR model of fluoroquinolone genotoxicity (pLOEC), both established by Zhao et al. [14], were used to evaluate the functional characteristics (plasma protein binding rate and genotoxicity) of 16 TRO derivatives (Table 2).

The plasma protein binding rates of 11 TRO derivatives decreased by different extents compared with that of trovafloxacin. The plasma protein binding rates of derivative-10 (1-C_2_H_5_-5-PH_2_-Trovafloxacin) and derivative-11 (1-C_3_H_7_-5-PH_2_-Trovafloxacin) decreased significantly by 24.18% and 20.60%, respectively. In addition, the genotoxicity (pLOEC) of the 11 TRO derivatives increased by different amounts compared to that of trovafloxacin (range of increase was 4.89–21.36%). Genotoxicity means that quinolones selectively inhibit two enzymes that play a role in DNA synthesis in bacteria, namely topoisomerases II and IV. This prevents replication, transcription, and repair of bacterial DNA, thus making it impossible for bacteria to grow and multiply. Therefore, increased genotoxicity can improve the medicinal effect of the drug.

In summary, the 11 TRO derivatives designed in this study have two beneficial effects on the human body, first they reduced the adverse effects (plasma protein binding rate), then they also enhanced the pharmacological effects (genotoxicity).

#### 3.3.2. Evaluation of Environmental Friendliness of TRO Derivatives Based on 3D-QSAR Model

The QSAR model of bioconcentration (log*K*_ow_) and photodegradation (log*t*_1/2_) of fluoroquinolones constructed by Zhao et al. [15,16] was used to predict the bioconcentration and photodegradation of 11 TRO derivatives screened here (Table 3).

The bioconcentration of 11 TRO derivatives decreased by 36.90–61.41%, while their photodegradability increased by 9.04–20.56%. The 11 TRO derivatives were less prone to enrichment than trovafloxacin in the environment, and the residual parts were more easily photodegraded.

#### 3.3.3. Evaluation of Stability of TRO Derivatives Based on Density Functional Theory

The binding rate to the plasma protein of 11 TRO derivatives designed by using the CoMSIA model decreased to different amounts, and these decreases were beneficial from the viewpoint of human health. To further achieve the derivatization of fluoroquinolones, it is necessary to characterise the stability (stability in the environment) of the derivatives and to evaluate the difficulty of the reaction.

The positive frequency value of a molecule can directly reflect whether the molecule can remain stable in the environment. When the positive frequency value of a molecule is greater than zero, it can remain stable in the environment; otherwise, it cannot [29]. Therefore, density functional theory was used to calculate the positive frequency values of 11 TRO derivatives to test their stability [30] (Table 4).

According to Table 4, the positive frequency values of all the 11 TRO derivatives are greater than zero, so the 11 derivatives designed herein can remain stable in the environment.

**Figure 3:**
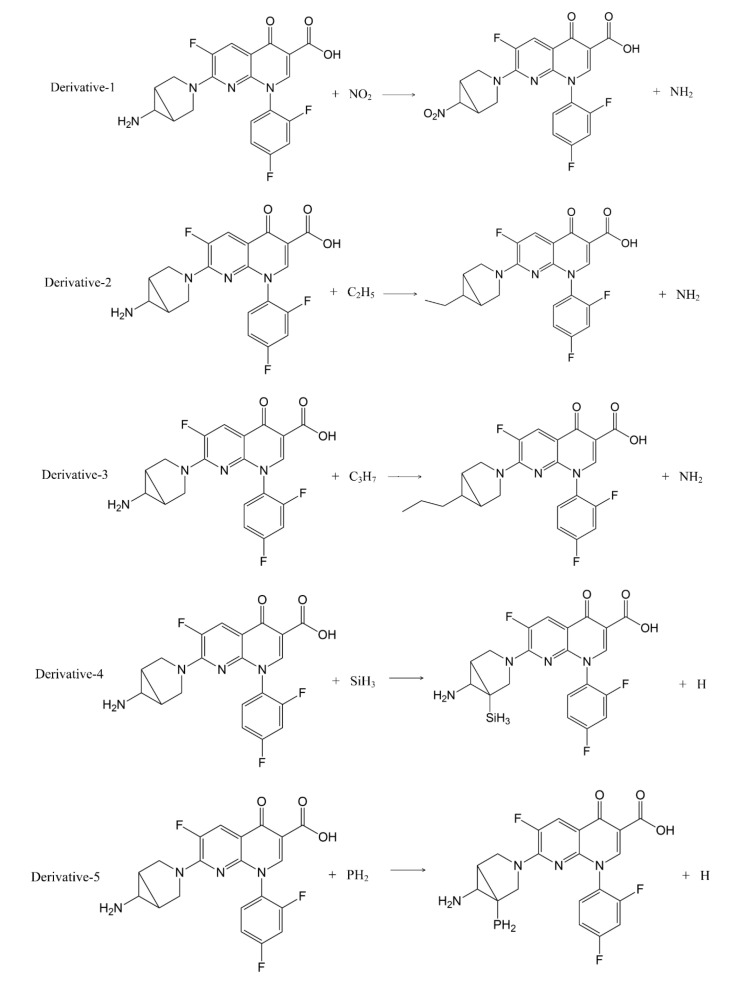

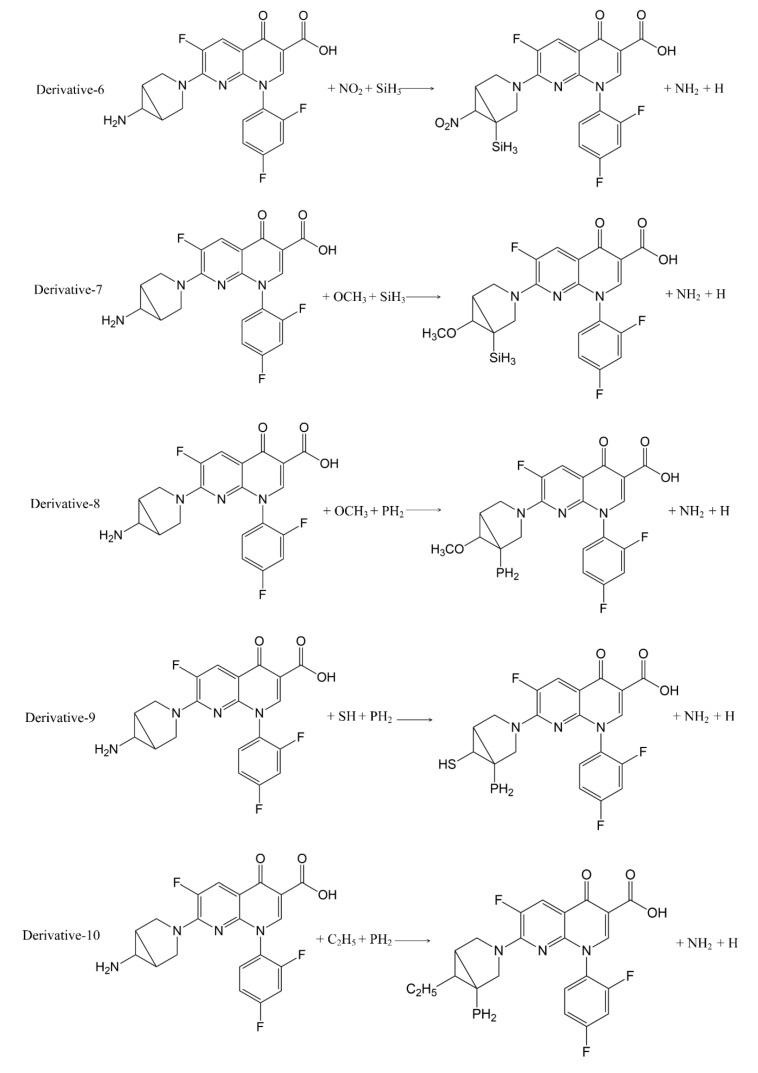

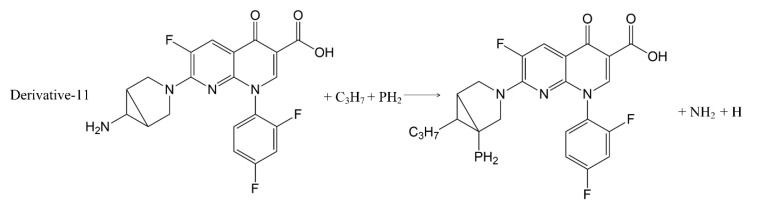
Substitution reaction paths of quinolone derivatives.

In addition to calculation of the positive frequency values of the TRO derivatives, the pathways of substitution reaction between the 7 substituents (-SH, -C_2_H_5_, -C_3_H_7_, -SiH_3_, -NO_2_, -PH_2_, -OCH_3_) and trovafloxacin were inferred (Figure 3). The Gibbs free energy change (ΔG) of the 11 substitution reactions were calculated using Formula (2) to judge the possibility of occurrence of the substitution reaction pathways [31,32] (Table 5).

(2) ΔG=∑G(Product)−∑G(Reactant)

The ΔG values of the 11 substitution reaction pathways were less than 0, indicating that the substitution reactions can proceed spontaneously, and that the inferred substitution reaction pathways were reasonable.

#### 3.3.4. ADMET Prediction of Trovafloxacin Derivatives

DS software was used to predict the absorption (A), distribution (D), metabolism (M), excretion (E) and toxicity (T) of TRO and its derivatives [33,34,35,36,37]. The aqueous solubility, human intestinal absorption, blood–brain barrier penetration, cytochrome P450 2D6 inhibition, hepatotoxicity and plasma protein binding rate were measured. Among them, the aqueous solubility of the molecule is closely related to the distribution and delivery of the drug in the body, and is one of the key factors for the preparation of a drug. Human intestinal absorption and blood–brain barrier can respectively influence the absorption of drugs from the intestines and enter the brain tissue to exert their drug effects. Cytochrome P450 is the main metabolic enzyme of drugs and other internal and external sources. Its activity may be inhibited or induced by drugs, which is essential for the metabolism of drugs in the body. In addition, the liver is an important organ for drug metabolism and is easily damaged by drugs; drugs will bind to plasma proteins at a certain ratio after being taken, so drugs are divided into bound and free types in the body, and only the free type has drug activity, that is, drugs are not combined with plasma protein [38].

Good drugs should have suitable aqueous solubility, good blood–brain barrier penetration, low toxicity and no inhibition of cytochrome P450, and mainly present the free type in plasma and have good intestinal absorption [39]. Through the prediction of TRO, derivative-10 and derivative-11, it was found that the 25 °C water solubility (the level is 2, low) and the human intestinal absorbability (the level is 0, good) of derivative-10 and derivative-11 relative to TRO were unchanged in the level. It showed that derivative-10 and derivative-11 have good solubility and intestinal absorption, and meet the standards of drug preparations. The blood–brain barrier penetration of the two derivatives was improved relative to TRO, hepatotoxicity was reduced, and there was no inhibitory effect on cytochrome P450 (Bayesian score was less than 0.162). However, the plasma protein binding rate of derivative-11 increased relative to TRO, while derivative-10 (level 0, good) remained unchanged. Therefore, among all derivatives, derivative-10 has the most characteristics of a new drug and is better than the target molecule TRO.

### 3.4. Analysis of Mechanism of Decrease in TRO Derivatives’ Plasma Protein Binding Rate Based on Molecular Docking

The target molecule and the 11 TRO derivatives were docked with plasma protein (ID:5NU7) by using Discovery Studio [40], and the relationship between the scoring function and the binding rate was further discussed (Table 6).

Except for derivative-1, the scores of the other fluoroquinolone derivatives were lower than that of trovafloxacin, and the level of decrease was consistent with the decrease in the plasma protein binding rate. For example, the plasma protein binding rates of derivative-10 and derivative-11 were 24.18% and 20.60% lower than that of trovafloxacin, respectively, and the corresponding docking results decreased by 24.43% and 17.83%. The above results further confirmed that the binding ability to plasma protein of the 11 TRO derivatives was reduced which compared with that of the target molecule.

#### 3.4.1. Qualitative Analysis of Mechanism of Decrease in Plasma Protein Binding Rate of TRO Derivatives Based on Changes in Amino Acid Residue

**Figure 4:**
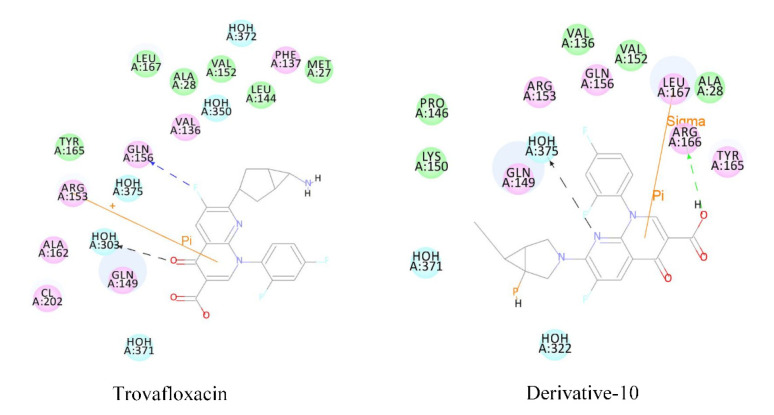
Molecular docking of trovafloxacin and derivative-10 with plasma protein (ID:5NU7).

Fluoroquinolones as antigens can bind to plasma proteins and stimulate the body to produce drug antigen-specific antibodies, thus reducing the number of white blood cells. The plasma protein binding rate and docking score of derivative-10 decreased significantly. Therefore, we subjected molecular docking results of derivative 10 to mechanism analysis mainly by studying the polarity and number of amino acid residues that play major roles in the acceptor molecule when derivative-10 binds to plasma protein (Table 7 and Figure 4).

When trovafloxacin was docked with plasma protein, the amino acid residues making interactions could be divided into two categories; hydrophobic amino acids represented by Leu, Val, Phe, Met, Ala, Tyr, and hydrophilic amino acids represented by Gln and Arg. There were nine hydrophobic amino acid residues and three hydrophilic amino acid residues. When derivative-10 was docked with plasma protein, the hydrophobic amino acids were mainly Leu, Val, Pro, Ala, and Tyr, that is, a total of six amino acid residues, while the hydrophilic amino acid residues were mainly Gln, Lys, and Arg, that is, a total of five amino acid residues. This indicated that when quinolones enter into plasma protein composed of hydrophilic amino acids, the interaction between the receptor protein and the donor molecule weakens, and the decrease in the docking function score and the plasma protein binding rate are related to changes in amino acid residues.

#### 3.4.2. Quantitative Analysis of the Decrease in Plasma Protein Binding Rate of TRO Derivatives

**Figure 5:**
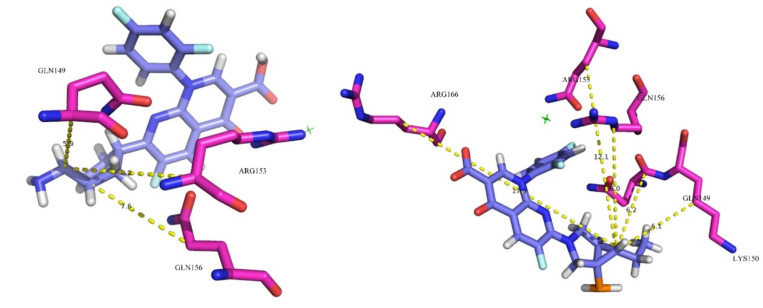
Binding conformation of trovafloxacin and derivative-10 with plasma protein ligand in binding domain.

To explore the effect of hydrophilicity on the binding force between derivative-10 and plasma protein, the distances between substitution site 1- of Trovafloxacin and derivative-10 and the hydrophilic amino acid residues at the active site were measured and averaged (Figure 5 and Table 8).

The bond lengths between the NH_2_ substituent at site 1 of trovafloxacin and the hydrophilic amino acid residues Gln149, Gln156, and Arg153 at the active site of the plasma protein were 5.9 Å, 7.6 Å, and 8.2 Å, respectively, and the average distance was 7.2 Å. The bond lengths between the C_2_H_5_ substituent at site 1 of derivative-10 and the hydrophilic amino acid residues Gln149, Gln156, Arg153, Arg166, and Lys150 at the active site of the plasma protein were 6.2 Å (>5.9 Å), 9.0 Å (>7.6Å), 12.1Å (>8.2 Å), 17.5 Å, and 6.1 Å, respectively, with an average distance of 10.2 Å. The binding rate and docking scoring function of trovafloxacin to plasma protein in Table 2 and Table 6 are higher than those of derivative-10. It was concluded that the further the average distance between the substituent of site 1- and the hydrophilic amino acid residue, the weaker is the hydrophilic effect between fluoroquinolone and plasma protein, and the smaller is the binding force between them. Therefore, the average distance between substituent 1- and the hydrophilic amino acid residue is negatively correlated with the binding force between fluoroquinolone and plasma protein, as well as with the plasma protein binding rate of fluoroquinolone.

The further the distance (average distance) between substitution site 1- of derivative-10 and the hydrophilic amino acid residues at the plasma protein binding site is, the more difficult it is to form a strong hydrophilic interaction between fluoroquinolone and plasma protein. Therefore, the weaker the binding force between fluoroquinolone and plasma protein, the lower is the plasma protein binding rate of fluoroquinolone. Thus, this study further explains the reason for the difference in binding between TRO derivatives and plasma protein.

### 3.5. Interaction Analysis between TRO Derivative and Plasma Protein Based on Molecular Dynamics Simulation

Molecular dynamics simulations [41,42] mainly employ the basic principles of Newtonian mechanics, taking molecular motion as the main simulation object and studying the dynamic process of the motion state of all particles in the system with time. Molecular dynamics simulations can provide detailed information about changes in protein structure with time. Moreover, the calculation results of molecular dynamics simulations are very similar to real experimental results in many aspects, and they can be used to test the rationality of statistical mechanics theory and to study the effect of microscopic forces on macroscopic properties. Since Mccammon et al. [43] first used molecular dynamics simulations to study the dynamic properties of bovine insulin inhibitors in 1977, this method has been used to study drugs, protein molecules, and in other fields [44,45,46].

#### 3.5.1. Analysis of Molecular Trajectories of TRO Derivatives Based on RMSD and RMSF

Molecular dynamics simulations of the complexes of trovafloxacin and derivative-10 with plasma protein were performed, and their trajectories were analysed based on root mean square deviation (RMSD) and root mean square fluctuation (RMSF).

**Figure 6:**
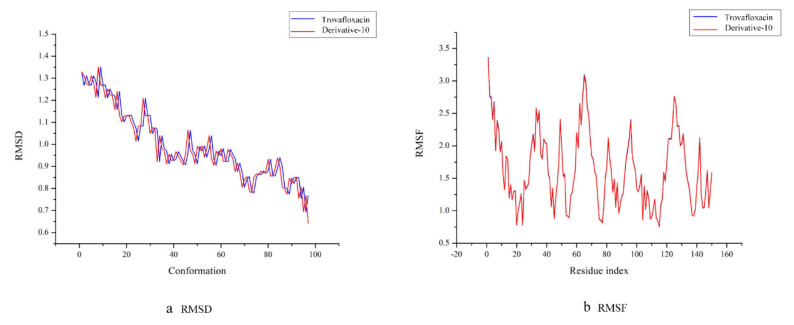
RMSD and RMSF curves of trovafloxacin and derivative-10 binding to plasma protein. (**a**): RMSD; (**b**): RMSF.

RMSD refers to the root mean square deviation of each atom between the protein conformation and its initial conformation. Each conformation has an RMSD value. The smaller the RMSD value is, the lighter is the motion amplitude of each atom in the protein and the higher is its stability [47]. Figure 6a shows that the RMSD trajectories of trovafloxacin and derivative-10 are essentially identical, which means that during molecular dynamics simulation, trovafloxacin and derivative-10 can be thought to have exhibited good stability. In addition, in the first 40 conformations, the motion trajectory showed a significant downward trend, and as a result, the trajectories of the complexes fluctuate greatly at this stage. When the simulation system reached the 40th conformation, the RMSD value of the entire system was low, and the fluctuation amplitude was small. As a result, all systems tended to be balanced at this time.

The RMSF value refers to the root mean square fluctuation of amino acid residues, which reflects the conformation of each amino acid residue in the molecular dynamic trajectory. A high RMSF value indicates a less stable conformation of amino acids and vice versa [48]. According to Figure 6b, the RMSF trajectories of trovafloxacin and derivative-10 with the plasma protein complexes are essentially identical. With a small number of amino acid residues, the RMSF value of trovafloxacin was slightly higher than that of derivative-10. Therefore, compared to trovafloxacin, derivative-10 can be considered more stable. Moreover, the amino acid residues of trovafloxacin and derivative-10 fluctuated widely in the initial stage, which might be caused by the fact that the interaction area at this stage was an unstable region of plasma protein. Then, exhibited a staged decline trend wax, indicating that the entire system tended to stable. The lowest fluctuation of amino acid residues occurred in the range of 100–120, indicating that the stability of the complexes was the highest in this stage.

#### 3.5.2. Qualitative and Semi-quantitative Analysis of the Molecular Trajectories Based on Potential Energy and Total Energy

**Figure 7:**
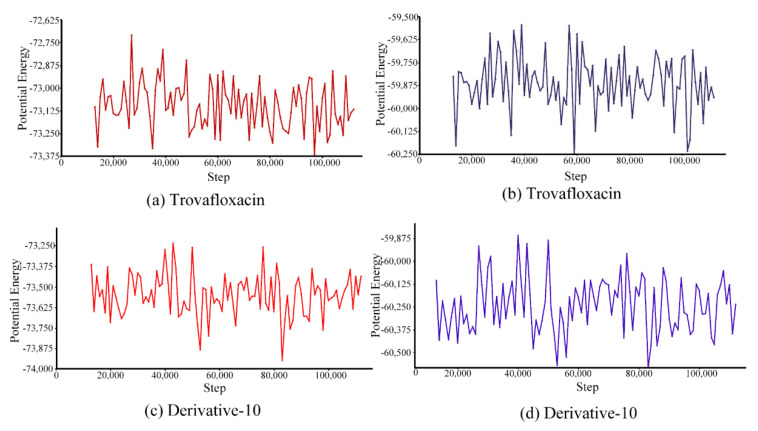
Potential energy and total energy trajectory of trovafloxacin and derivative-10 binding to plasma protein. (**a**): potential energy of trovafloxacin; (**b**): total energy of trovafloxacin; (**c**): potential energy of derivative-10; (**d**): total energy of derivative-10.

Energy simulation was performed for the complexes of trovafloxacin and derivative-10 with plasma protein (Figure 7).

Because of the interaction force between molecules, energy is related to their relative positions, namely molecular potential energy and total energy [49]. The motion trajectory of potential energy (Figure 7a,c) showed that the potential energy of trovafloxacin varied between −73,375 kcal/mol and −72,625 kcal/mol, and its average potential energy was approximately −73,100 kcal/mol. However, the potential energy of derivative-10 ranged from −73,875 kcal/mol to −73,250 kcal/mol, and the average potential energy was approximately −73,500 kcal/mol. Therefore, the range of potential energy and average potential energy of the combination of derivative-10 and plasma protein were lower than those of trovafloxacin combined with plasma protein. This result indicates that the interaction between derivative-10 and plasma proteins was weaker.

In addition to studying changes in the potential energy of trovafloxacin and derivative-10, we further analysed the total energy in the simulated system (Figure 7b,d). The total energy of trovafloxacin combined with plasma protein varied between −60,250 kcal/mol and −59,500 kcal/mol, and the average total energy was approximately −59,875 kcal/mol. The total energy change of derivative-10 after binding to plasma protein ranged from −60,500 kcal/mol to −59,875 kcal/mol, with an average total energy of approximately −60,250 kcal/mol. Therefore, the total energy variation range and the average total energy of derivative-10 were lower than those of trovafloxacin.

The interaction energy between derivative-10 and plasma protein was lower than that between trovafloxacin and plasma protein in the molecular dynamics simulation system, further explained the decrease in the binding rate of trovafloxacin derivatives to plasma proteins.

## 4. Conclusions

Based on the 3D-QSAR model, a variety of environmentally friendly trovafloxacin derivatives with low plasma protein binding rates were designed in this study. Moreover, the density functional theory, molecular docking, and molecular dynamics simulation methods were used to analyse the reason for the decrease in the plasma protein binding rate of trovafloxacin derivatives. In this manner, the findings of this study can provide theoretical support for discovering novel antibiotic drug molecules that cause little harm to the environment and human health.

## Figures and Tables

**Table 1:** Experimental and model-predicted value of Log*f*_b_.

No.	Compounds	Experiment	Predicted	Relative Error (%)
1 ^a^	Nalidixic acid	1.968	1.966	−0.13%
2 ^a^	Oxolinic Acid	1.886	1.893	0.35%
3 ^a^	Cinoxacin	1.833	1.830	−0.14%
4 ^b^	Rosoxacin	1.908	1.871	−1.96%
5 ^a^	Norfloxacin	1.415	1.417	0.14%
6 ^a^	Enoxacin	1.544	1.551	0.45%
7 ^a^	Ciprofloxacin	1.362	1.364	0.17%
8 ^a^	Enrofloxacin	1.544	1.526	−1.17%
9 ^a^	Difloxacin	1.531	1.536	0.30%
10 ^a^	Temafloxacin	1.447	1.438	−0.63%
11 ^b^	Ofloxacin	1.398	1.623	16.10%
12 ^a^	Levofloxacin	1.380	1.394	1.00%
13 ^b^	Rufloxacin	1.785	1.708	−4.33%
14 ^a^	Fleroxacin	1.462	1.457	−0.37%
15 ^a^	Sparfloxacin	1.362	1.362	0.02%
16 ^a^	Trovafloxacin	1.748	1.749	0.05%

^a^ Training set; ^b^ Test set.

**Table 2:** Predicted results of log*f*_b_ and pLOEC of TRO derivatives.

No.	Structure	log*f*_b_ Pred.	*f*_b_ Pred.	Relative Error (%)	pLOEC	Relative Error (%)
Derivative-1	1-NO_2_-Trovafloxacin	1.738	54.702	−2.32%	8.102	4.89%
Derivative-2	1-C_2_H_5_-Trovafloxacin	1.738	54.702	−2.32%	8.203	6.20%
Derivative-3	1-C_3_H_7_-Trovafloxacin	1.724	52.966	−5.42%	8.445	9.33%
Derivative-4	5-SiH_3_-Trovafloxacin	1.722	52.723	−5.85%	9.374	21.36%
Derivative-5	5-PH_2_-Trovafloxacin	1.721	52.602	−6.07%	9.166	18.67%
Derivative-6	1-NO_2_-5-SiH_3_-Trovafloxacin	1.746	55.719	−0.50%	8.830	14.32%
Derivative-7	1-OCH_3_-5-SiH_3_-Trovafloxacin	1.735	54.325	−2.99%	9.008	16.62%
Derivative-8	1-OCH_3_-5-PH_2_-Trovafloxacin	1.736	54.450	−2.77%	8.650	11.99%
Derivative-9	1-SH-5-PH_2_-Trovafloxacin	1.733	54.075	−3.44%	8.856	14.66%
Derivative-10	1-C_2_H_5_-5-PH_2_-Trovafloxacin	1.628	42.462	−24.18%	8.594	11.26%
Derivative-11	1-C_3_H_7_-5-PH_2_-Trovafloxacin	1.648	44.463	−20.60%	8.821	14.20%
Derivative-12	1-H-Trovafloxacin	1.778	59.979	7.11%	7.684	−0.52%
Derivative-13	1-CH_3_-Trovafloxacin	1.769	58.749	4.91%	8.023	3.87%
Derivative-14	1-C_2_H_3_ -Trovafloxacin	1.756	57.016	1.82%	8.061	4.36%
Derivative-15	1-OCH_3_ -Trovafloxacin	1.772	59.156	5.64%	8.012	3.73%
Derivative-16	1-SH -Trovafloxacin	1.770	58.884	5.15%	8.156	5.59%

**Table 3:** Predicted log*K*_ow_ and log*t*_1/2_ values of quinolone derivatives using QSAR models.

No.	log*K*_ow_	Relative Error (%)	log*t*_1/2_	Relative Error (%)
Trovafloxacin	2.436		2.267	
Derivative-1	0.940	−61.41%	1.801	−20.56%
Derivative-2	1.537	−36.90%	1.994	−12.04%
Derivative-3	1.226	−49.67%	1.819	−19.76%
Derivative-4	1.319	−45.85%	2.001	−11.73%
Derivative-5	1.173	−51.85%	1.988	−12.31%
Derivative-6	0.991	−59.32%	1.806	−20.34%
Derivative-7	1.490	−38.83%	2.031	−10.41%
Derivative-8	1.412	−42.04%	2.062	−9.04%
Derivative-9	1.396	−42.69%	2.006	−11.51%
Derivative-10	1.166	−52.13%	2.023	−10.76%
Derivative-11	1.182	−51.48%	2.032	−10.37%

**Table 4:** Positive frequency calculations of quinolone derivatives.

No.	Frequency Value (cm^−1^)	No.	Frequency Value (cm^−1^)
Derivative-1	15.16	Derivative-7	14.6
Derivative-2	13.77	Derivative-8	15.14
Derivative-3	13.79	Derivative-9	15.29
Derivative-4	18.05	Derivative-10	9.69
Derivative-5	14.25	Derivative-11	14.81
Derivative-6	18.65		

**Table 5:** Gibbs free energy change (ΔG) of quinolones’ substitution reaction paths.

Reaction Paths	Gibbs Free Energy/(a.u.)		ΔG/(kcal·mol^−1^)
	For Reactants	For Products	
Path 1	−1704.0287	−1720.0301	−10,041.0544
Path 2	−1578.1252	−1594.1148	−10,033.6265
Path 3	−1617.4133	−1633.4027	−10,033.5255
Path 4	−1790.2267	−1806.2479	−10,053.4834
Path 5	−1841.3928	−1858.0118	−10,428.5490
Path 6	−1995.2549	−2011.2238	−10,020.6509
Path 7	−1905.2647	−1921.2198	−10,011.9674
Path 8	−1956.4308	−1972.9852	−10,388.0100
Path 9	−2240.1455	−2256.6822	−10,376.9232
Path 10	−1920.5175	−1937.0769	−10,391.2134
Path 11	−1959.8056	−1976.3648	−10,391.0352

**Table 6:** Docking results of target molecule and TRO derivatives with plasma protein.

No.	Libdock Scores	Relative Error (%)
Trovafloxacin	74.7975	
Derivative-1	81.1813	8.53%
Derivative-2	65.8328	−11.99%
Derivative-3	67.9220	−9.19%
Derivative-4	66.0899	−11.64%
Derivative-5	63.7173	−14.81%
Derivative-6	64.7691	−13.41%
Derivative-7	66.6251	−10.93%
Derivative-8	68.0351	−9.04%
Derivative-9	64.7427	−13.44%
Derivative-10	56.5253	−24.43%
Derivative-11	61.4623	−17.83%

**Table 7:** The number of hydrophilic/hydrophobic amino acid residue that trovafloxacin and derivative-10 bound to plasma protein.

Amino Acid Residues	Character	Number of Amino Acid Residues	
		Trovafloxacin	Derivative-10
Leu	hydrophobic	2	1
Val	hydrophobic	2	2
Phe	hydrophobic	1	0
Met	hydrophobic	1	0
Ala	hydrophobic	2	1
Tyr	hydrophobic	1	1
Gln	hydrophilic	2	2
Arg	hydrophilic	1	2
Lys	hydrophilic	0	1
Pro	hydrophobic	0	1

**Table 8:** Distances between trovafloxacin and derivative-10 and hydrophilic amino acid residues at the plasma protein binding site.

Compounds	Hydrophilic Amino Acid Residues	Distance from 1-Substituent (Å)	Average Distance (Å)
Trovafloxacin	Gln149	5.9	7.2
	Gln156	7.6	
	Arg153	8.2	
Derivative-10	Gln149	6.2	10.2
	Gln156	9.0	
	Arg153	12.1	
	Arg166	17.5	
	Lys150	6.1	
